# Editorial: Realizing universal health coverage in India

**DOI:** 10.3389/fpubh.2023.1243676

**Published:** 2023-07-27

**Authors:** Nerges Mistry, Sandhya Venkateswaran, Rama Baru, Vikram Patel

**Affiliations:** ^1^The Foundation for Medical Research, Mumbai, India; ^2^Centre for Social and Economic Progress, New Delhi, India; ^3^Lancet Citizen's Commission on Reimagining India's Health System, New Delhi, India; ^4^Centre of Social Medicine and Community Health, Jawaharlal Nehru University, New Delhi, India; ^5^Department of Global Health and Social Medicine, Harvard Medical School, Boston, MA, United States; ^6^Department of Global Health and Population, Harvard T.H. Chan School of Public Health, Boston, MA, United States

**Keywords:** civil society, health insurance, India, out-of-pocket expenses, universal health coverage

Universal Health Coverage (UHC) is a full basket with ambitious goals for its 5 pillars, viz., finance, human resources for health, citizen engagement, governance, and technology for the articulation of a road map. While the goals of UHC are largely common, the pathways leading to goal realization may vary across countries. India, a federation of 28 States and 8 union territories, has defined roles in healthcare ascribed to the Central and State Governments played out on the basis of their relationships. There also exists a heterogenous minimally regulated private sector, ranging from formal providers of both the allopathic and traditional systems of medicine, as well as informal providers that are often the first port of call for patients in underserved areas. Health care under the Indian Constitution is primarily a State subject, though the Center has a primary role in National Disease Control Programmes, Reproductive and Child Health, National Health Mission, and a national medical insurance programme for lower income groups. There is in the recent past an increasing trend shift toward public private partnerships in healthcare (1) given that a majority of people first seek care from private providers. However, the quality of healthcare provided is extremely variable given the lack of regulations and oversight resulting in non-adherence to established clinical guidelines (2).

The 5 articles in this Research Topic on UHC in India, attempt to address, albeit partially, the complex issues that underline the journey to UHC. However, the majority of the manuscripts of the issue focus on health financing and risk pooling. This remains a key issue given India's low public expenditure on health which stands at 2–1.6% of GDP, a level below other countries in the region (3). However, out of pocket expenses (OOPE) at point of service remain consistently high that increases economic vulnerability, in that health expenditure becomes the second largest cause of indebtedness. While the need to increase public health, spending is constant in recent years, there has been growing focus on the approaches of pooled funds and demand side financing (4).

The theory of change roadmap laid out by Chaudhuri et al. was steered by the Lancet Citizen's Commission toward achieving UHC in India in the next decade. While consensus on the approach was achieved in the areas of citizen centricity, efficient use of public finances, shift to team-based care management, the use of appropriate technology, debates continue in the areas of financing service delivery, and accountability. Overall, a transformational change is required in the Indian healthcare system with emphasis on role clarity of human resources at each level including local government structures, reliable supply of drugs, diagnostics and transport for patients, POC equipment, and patient/environmental samples. Not much has been mentioned about the new disease agents which will fashion health systems substantially in the coming years thanks to climate change and deteriorating ecosystems. In the face of these newer emerging threats, human competencies particularly at the primary and secondary level of healthcare need to be built.

The paper by Ashraf et al. in these series identifies pathways that can bring consumers to the insurance market and improve relationships between the commercial health insurance providers and their lay clients now fraught with mistrust and concealment of risk on the part of the users and the lack of power of the insurers on the largely unregulated private sector providers engaged in irrational/excessive testing and treatment. They opine that quality care could be better provided through an integration of care and insurance through incentive alignment. This places reliance on vigorous primary care with emphasis on preventive screening and awareness generation which goes beyond illness. This is laudable except that it goes against a) behavioral norms of Indians with regard to their aversion to frequent screening, and mistrust of the quality of care (5) b) non-operability in rural populations with minimal contact between providers and commercial insurance c) ignoring the non-medical drivers of ill health in the ecosystem (6, 7). Offering consistently reliable quality of curative care is the first step toward minimization of risk of severe disease by encouraging people to seek help early for their symptoms from a health system they trust. A government financed insurance system largely for in-patient care is also recently being played out in its myriad forms across states (8, 9) but the design needs more attention to derive greater value including strategic and practical purchasing norms including costs of outpatient care, treatment and diagnostics (10). Costing expertise in India, however, needs upgrading to mitigate inconsistencies, build local capacity and link costing to public budgeting (11).

The conclusion drawn by the analysis undertaken by Mor is that irrespective of whether OOPE or pooled risk is adopted by any country, it must invest first in public merit goods viz. immunizations and strong comprehensive primary health care that can result in low DALY rates even at low levels of health expenditures. A second conclusion draws attention to quality of healthcare provided, which if not achieved, could result in poor outcomes even with high levels of pooling or OOPE. Lastly of course the article lays value on the production of good health which UHC should focus on using the one health approach which includes social/environmental drivers of ill health.

The role of civil society in building primary level human capacities that can link with the urban health system has been highlighted in the perspective by Jayaraman and Fernandez. This is not a new observation, since historically pioneering initiatives for coverage to the most vulnerable section of our rural populations has been amply demonstrated since the 1960s. The lessons need to be extended now to our urban populations which have their own set of distinct health problems. Civil society can therefore catalyze innovations and processes in the field but their translation on a wider plane requires the right transponders at the policy level with aligning beliefs and reach to transform experiments into systems.

The paper by Venkateswaran et al. asks a pertinent question as to how do you create incentives for the political system to invest in health as a priority. While seeking political legitimacy or displaying a new ideology, a new regime may prioritize health. Its sustainability however should rest on the initiatives of citizens and the voluntary sector, by speaking truth to power in the creation of health-related evidence thereby contributing to the right type of legitimacy. Indeed, the voluntary sector in India has historically contributed both to the concept and operationalization of a community-based health care system that evolved to the National Health Mission (12). Notably, this contribution was backed by a political coalition and synthesis of opinion between the centrist and left parties in India.

The UHC agenda is moving along slowly despite multisectoral efforts including increasing reliance on third party payment systems, emergence of digital health (13) and the sporadic Right to Health Act at state level. A lackadaisical HSG approach, insufficient funds, inadequate health force and weak regulation and policies due to Center-State anomalies are challenges to achievement of UHC. These have hindered the meeting of needs of India's massive and diverse population. There is also little to substantiate that health is an electoral priority for Indian citizens and whether the value of overall wellbeing and health care is a part of India's growth trajectory. The choices underlying furthering of health vis-à-vis industrial development goals are all too apparent whether related to reduction in use of coal or loss of virgin forest cover (14). Deepening our understanding of factors that affect policy reforms is important (15) but we should not overlook the existence of basic values of compassion, and understanding of human agency. Health is not value neutral healthcare.

## Author contributions

The Editorial was framed by NM with inputs from SV, RB, and VP. All authors contributed to the article and approved the submitted version.
